# Effects of antidepressant on *FKBP51* mRNA expression and neuroendocrine hormones in patients with panic disorder

**DOI:** 10.1186/s12888-024-05704-4

**Published:** 2024-04-10

**Authors:** Zhili Zou, Yulan Huang, Michael Maes, Jinyu Wang, Ying He, Wenjiao Min, Bo Zhou

**Affiliations:** Provincial Center for Mental Health, Sichuan Academy of Medical Sciences & Sichuan Provincial People’s Hospital, 610072 Chengdu, China

**Keywords:** Panic disorder, HPA axis, Neuroendocrine hormone, Escitalopram

## Abstract

**Objective:**

The purpose of this study was to investigate the effects of escitalopram on the peripheral expression of hypothalamic-pituitary-adrenal (HPA) axis-related genes (*FKBP51*, *HSP90*, *NR3C1* and *POMC*) and HPA-axis hormones in patients with panic disorder (PD).

**Methods:**

Seventy-seven patients with PD were treated with escitalopram for 12 weeks. All participants were assessed for the severity of panic symptoms using the Panic Disorder Severity Scale (PDSS). The expression of HPA-axis genes was measured using real-time quantitative fluorescent PCR, and ACTH and cortisol levels were measured using chemiluminescence at baseline and after 12 weeks of treatment.

**Results:**

At baseline, patients with PD had elevated levels of ACTH and cortisol, and *FKBP51* expression in comparison to healthy controls (all *p* < 0.01). Correlation analysis revealed that *FKBP51* expression levels were significantly positively related to cortisol levels and the severity of PD (all *p* < 0.01). Furthermore, baseline ACTH and cortisol levels, and *FKBP51* expression levels were significantly reduced after 12 weeks of treatment, and the change in the PDSS score from baseline to post-treatment was significantly and positively related to the change in cortisol (*p* < 0.01).

**Conclusions:**

The results suggest that PD may be associated with elevated levels of ACTH and cortisol, and *FKBP51* expression, and that all three biomarkers are substantially decreased in patients who have received escitalopram treatment.

**Supplementary Information:**

The online version contains supplementary material available at 10.1186/s12888-024-05704-4.

## Introduction

Panic disorder (PD) is the most common anxiety disorder characterized by recurrent and unexpected panic attacks. The estimated lifetime prevalence of PD is approximately 3.7% [1, 2]. PD often appears after exposure to acute or chronic stress, such as childhood trauma or adverse life events [3–5] and may cause alterations in response to stress. The hypothalamic-pituitary-adrenal (HPA) axis is the main neuroendocrine system that controls stress responses, and the glucocorticoid receptor (GR) is the most important regulator of the negative feedback system of the HPA axis negative feedback system [6]. FK-506 binding protein 5 (FKBP5) is a co-chaperone of heat shock protein 90 (Hsp90) that regulates NRC31 activity via a short negative feedback loop [7]. Pro-opiomelanocortin (POMC) is the archetypal polypeptide precursor of hormones and neuropeptides [8], which is a precursor of adrenocorticotropic hormone (ACTH). Furthermore, previous research found that the variation and methylation of the *NR3C1*, *FKBP51*, *Hsp90* or *POMC* gene is involved in the pathophysiology of many psychiatric disorders and with abnormal gene expression or neuroendocrine hormones [9–14]. Furthermore, hyperactivity of the HPA axis has been observed in patients with PD [15], and dysfunction of the HPA axis in patients with PD is among the most robust biological findings.

Selective serotonin reuptake inhibitors (SSRIs) are recommended as first-line treatment for PD, especially escitalopram. SSRIs may act, in part, by modulating the HPA axis [15], whose alteration could be partially responsible for treatment efficacy. For example, animal studies have found that escitalopram decreased the expression of corticotropin-releasing factors in the hippocampal while increasing the hypothalamic and hippocampal expression of GR [16]. Four weeks of intervention with escitalopram decreased the relative change from baseline in the expression of GRα mRNA compared with placebo [17]. SSRI treatment reduced salivary cortisol in patients with major depression to levels of controls, and cortisol reduction was significantly correlated with improvement in depressive symptoms [18]. SSRI treatment of generalized anxiety disorder (GAD) in older adults reduces HPA axis hyperactivity [19]. Research evidence from our previous work shows higher serum levels of ACTH and cortisol in PD patients [20]. It is speculated that antidepressants may affect the expression of genes related to the HPA axis and neuroendocrine hormones in patients with PD. However, the treatment effects of escitalopram on the HPA axis function in PD have not yet been reported.

Therefore, the main objective of this study was to investigate changes in peripheral expression of the *NR3C1*, *FKBP51*, *Hsp90* and *POMC* genes, plasmalevels of ACTH, and cortisol in patients with PD before and after treatment.

## Methods

### Study sample

We recruited 77 patients with PD from inpatient and outpatient populations in the psychosomatic department, Sichuan Provincial People’s Hospital. 71 patients with PD completed a 12-week follow-up. The primary diagnosis of PD was based on the patient version of the structured clinical interview for the Diagnostic and Statistical Manual of Mental Disorders, Fourth Edition (DSM-IV) (SCID-1) [21], which was performed by trained psychiatrists. All patients were first diagnosed and current with PD and none of the patients had any other psychiatric disorders. All PD patients were drug naive and had no history of taking antidepressants or other psychotropic drugs. In addition, 82 volunteers were recruited as healthy controls (HCs) in this study. The SCID was administered by a trained clinical psychiatrist to exclude lifetime or current diagnoses of PD and any other psychiatric disorders in these volunteer controls.

All subjects had no acute or chronic infection, autoimmune disorders, allergies, and other somatic disorders and had a body mass index (BMI) between 18 and 25. All patients were free of anti-inflammatory, immunosuppressive, contraceptives, or alcohol consumption within 2 weeks prior to examination. All participants were Han Chinese living in China. All experiments were carried out according to the Declaration of Helsinki of the WMA. This study was approved by the Ethics Committee of the Sichuan Provincial People’s Hospital (2018.16). All participants provided written informed consent before the initiation of study procedures.

### Clinical assessment

All participants were assessed for the severity of panic symptoms using the Panic Disorder Severity Scale (PDSS). The PDSS comprises seven items, and participants are instructed to rate each item from 0 (none) to 4 (extremely severe), based on the severity of each symptom; higher questionnaire scores indicate higher disease severity [22]. The Chinese version of PDSS (PDSS-CV) has good internal consistency (Cronbach’s alpha) with an overall score of 0.83 [23].

### Treatment

All patients received treatment for 12 weeks with escitalopram (10–20 mg qd), other psychotropic drugs were not allowed during the study. The patients were assessed at baseline and after 12 weeks of treatment, using PDSS, respectively.

### Measurement of ACTH and cortisol

Blood samples were collected from all participants while in a fasting state via venepuncture between 7:00 a.m. and 8:30 a.m. ACTH and cortisol levels were assessed at a single time point (8:00 to 9:00 am). Plasma levels of ACTH and cortisol were measured using chemiluminescence (IMMULITE 2000) according to the manufacturer’s instructions at the beginning of the study and after 2 weeks of treatment, respectively.

### Real-time fluorescent quantitative PCR

RNA was extracted from blood samples with a miRNeasy Mini Kit(50) (Qiagen, Germany) according to the manufacturer’s protocol. The Glyceraldehyde-3-phosphate dehydrogenase (GAPDH) gene was chosen as the housekeeping gene, the forward and reverse primers were designed by Shanghai Yingjun Biotechnology Company (Table S1). The reverse transcription was then performed in 16 µL reaction volumes using a HiScript Q RT SuperMix kit for the qPCR kit (Vazyme, Nanjing, China). RT-qPCR reaction using NuHi Robustic SYBR Green Mix (Xinhai, Suzhou, China). Finally, the PCR products were confirmed by melting curve analysis, and the melting curve of *NR3C1, FKBP51, HSP90 and POMC* is unimodal (Fig. S1, S2, S3, S4). GAPDH was used as a reference gene. Relative changes for target genes were determined after normalization to the expression of GAPDH. The expression levels of the mRNAs were calculated from the threshold cycle (Ct) value and the relative expression levels were calculated using the 2^−ΔCt^ method.

### Statistical analysis

Data analysis was performed using the Statistical Package for the Social Sciences (SPSS, version 29) and Statistica 12.0. The Kolmogorov-Smirnov test was used to determine the distribution of the data. Student’s t-test or the Mann-Whitney U test were used to check intergroup differences in continuous variables, and Pearson’s chi-square test was used for categorical variables. If the data was not normally distributed, the results were described as median (P25–P75). Pearson or Spearmen correlation analyses were used to assess associations between study variables in patients with PD. We used multiple regression analysis, the manual method, to delineate the effects of the changes in biomarkers (explanatory variables) on the changes in the dependent variable (the PDSS score). In addition, we entered (forced entry) possible confounders, including age, sex, BMI, drinking and smoking, to adjust for possible effects. We interpreted the effects of changes of the biomarkers on the changes in PDSS by evaluating standardized β coefficients, t-statistics, and exact p-values for each of the explanatory variables. Furthermore, we used false discovery rate(FDR) p correction to adjust for multiple comparisons or associations [24]. All the regression analyses were checked for collinearity and heteroskedasticity, using tolerance and the variance inflation factor, and the White and modified Breusch-Pagan test, respectively. We used GEE (generalized estimating equation), repeated measures analysis, to assess the effects of treatment on the PDSS score and the biomarkers. The pre-specified GEE, repeated measures design, included fixed categorical effects of time (treatment from baseline to 12 weeks later), while controlling for the effects of age, sex, BMI, and drinking and smoking behavior. This method allowed us to control for the effects of relevant covariates when examining treatment effects at the subject level. Logarithmic transformations were used where needed. For all analyses, statistical tests were two-tailed, and an alpha level of 0.05 was used to define statistical significance.

## Results

### Demographic and clinical characteristics

A total of 77 patients with PD (30 men and 47 women) and 82 HC (29 men and 53 women) were recruited. The mean age of the PD group was 35.08 ± 7.76 years, and the mean age of the control group was 36.04 ± 6.48 years. No statistically significant group differences were observed between patients with PD and HC in terms of age and sex (all *p* > 0.05). Furthermore, there was no significant difference in resident location, smoking history, drinking, and BMI in either group (all *p* > 0.05) (Table 1).

Furthermore, ACTH, cortisol levels, and *FKBP51* expression were higher in patients with PD than in HC at baseline(all *p* < 0.01), and the results remained significant after FDR multiple comparison adjustment (all *p* < 0.05). However, no statistically significant group difference was observed between PD patients and HC in the expression of *NR3C1*, *HSP90* and *POMC* at baseline (all *p* > 0.05) (Table 2).

### Correlation of the expression of genes related to the HPA axis at baseline with clinical manifestations

Spearman’s correlation analysis revealed that *FKBP51* expression levels were significantly positively related to cortisol level and severity of PD (all *p* < 0.01), the *NR3C1* expression levels were significantly positively related to the severity of PD (*p* < 0.01). ACTH levels were significantly positively related to cortisol levels (*p* < 0.01). However, no correlation was found between *HSP90*, *POMC* expression, ACTH, cortisol levels and the severity of PD (all *p* > 0.05) (Table 3).

### Changes in PDSS, HPA axis-related gene expression, ACTH, and cortisol levels from baseline to 12 weeks later

**Fig. 1:**
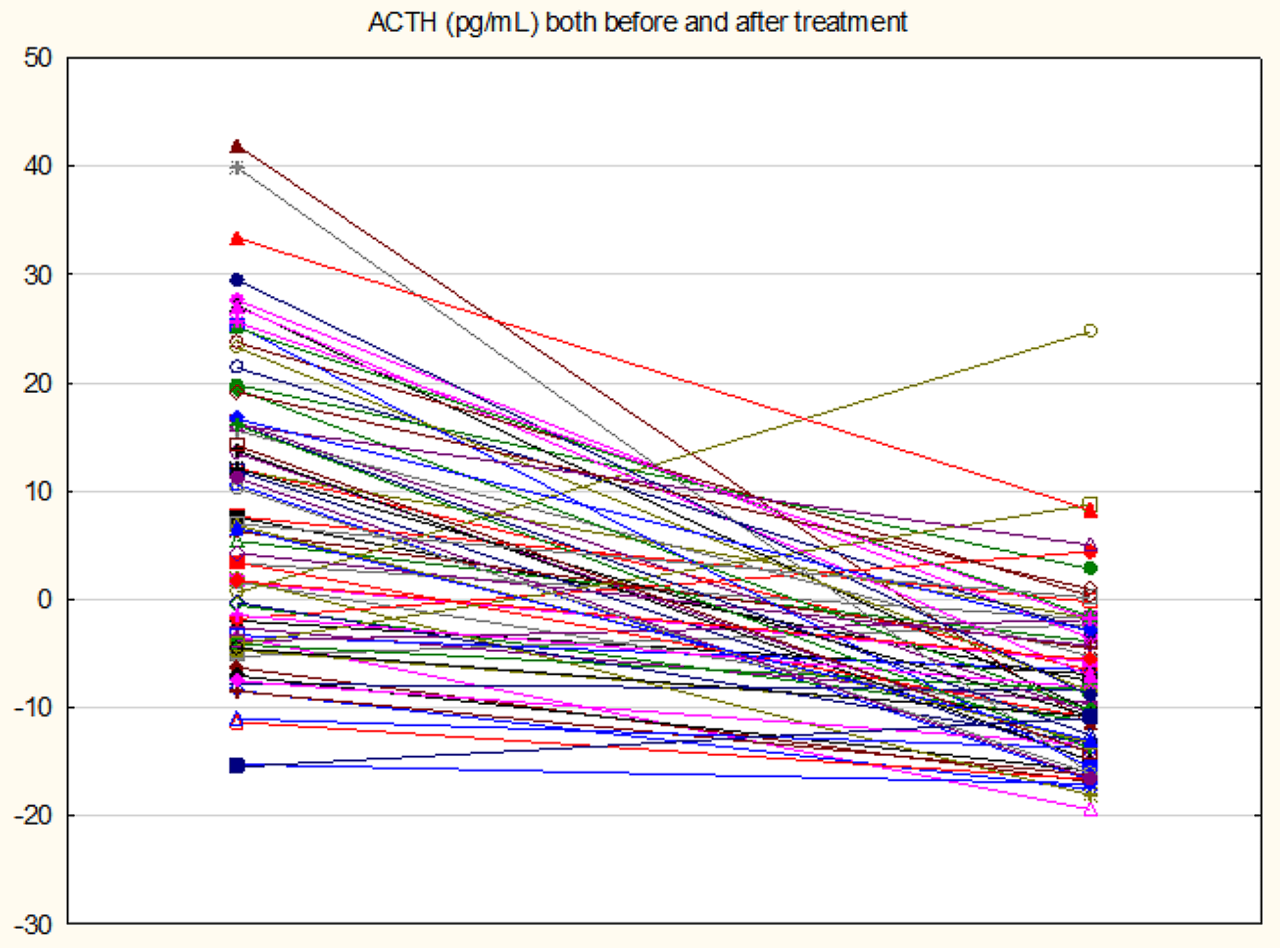
A Line plot showing the effects of 12 weeks treatment with escitalopram on ACTH levels in patients with panic disorder. Shown are the residualized ACTH values computed after adjusting for age, sex, body mass index, smoking and drinking behavior

**Fig. 2:**
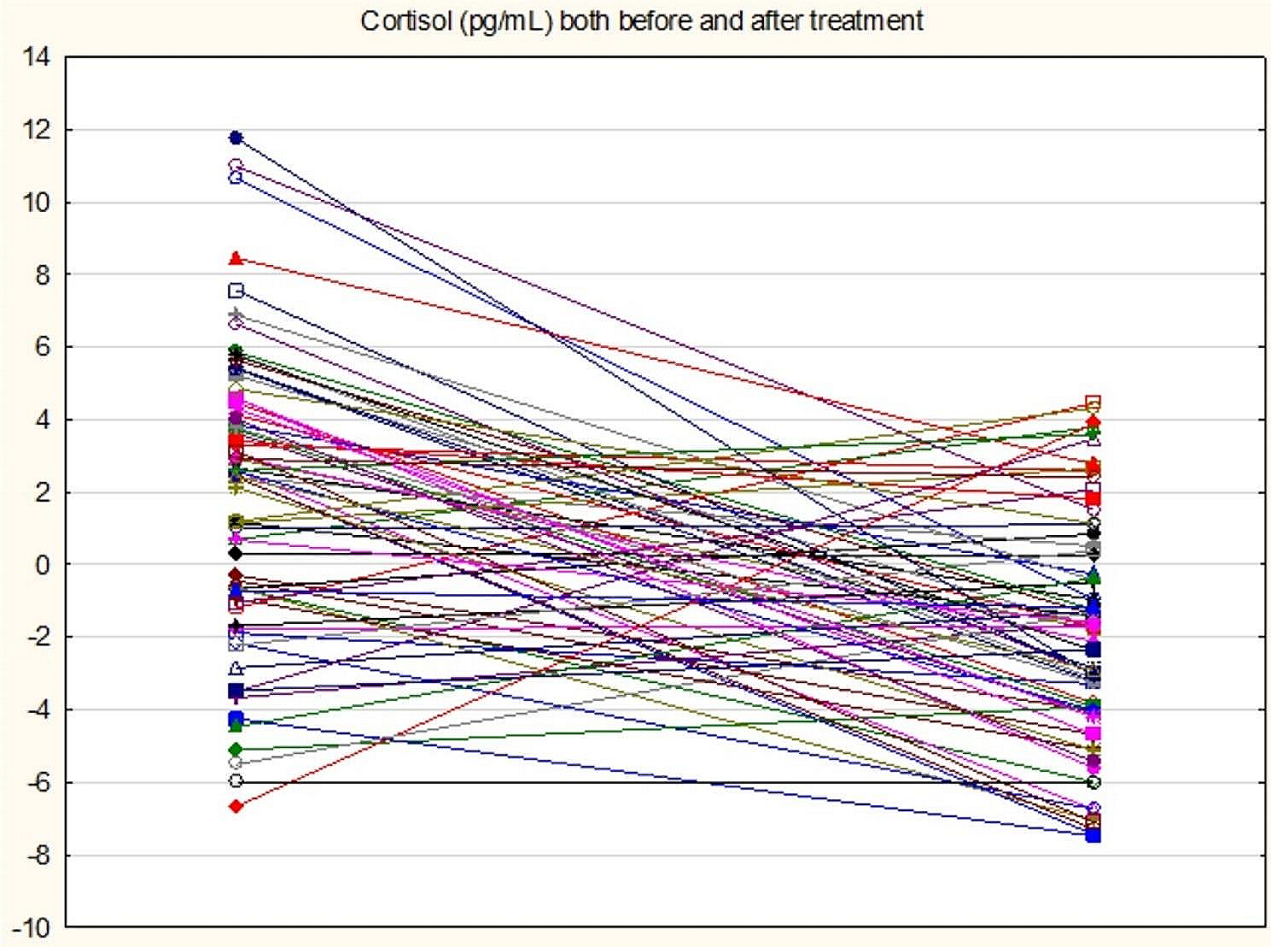
Line plot showing the effects of 12 weeks treatment with escitalopram on cortisol levels in patients with panic disorder. Shown are the residualized cortisol values computed after adjusting for age, sex, body mass index, smoking and drinking behavior

**Fig. 3:**
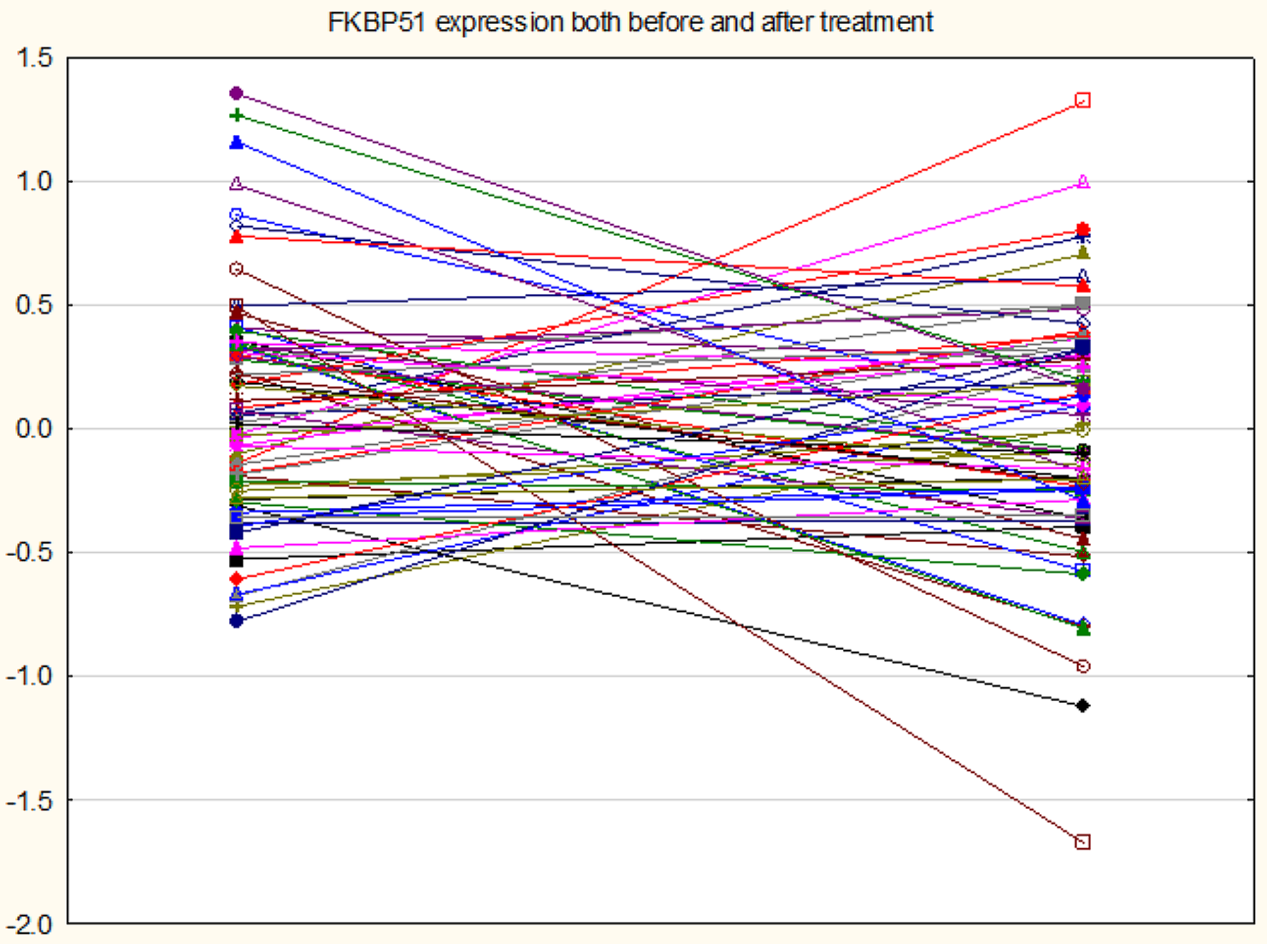
Line plot showing the effects of 12 weeks treatment with escitalopram on *FKBP51* expression in patients with panic disorder. Shown are the residualized log10 FKBP51 values computed after adjusting for age, sex, body mass index, smoking and drinking behavior

71 patients with PD completed the 12-week treatment with escitalopram. Table 4 shows the effects of 12-week treatment with escitalopram on the PDSS scores and all biomarkers. These data were analysed using GEE, repeated measurement design. All results were adjusted for possible effects of age, sex, BMI, drinking and smoking. The p values were FDR p-corrected. We found that the PDSS score, *FKBP51* expression, ACTH, and cortisol levels were significantly lowered in patients who have received escitalopram treatment. Age, sex, drinking and smoking did not have any significant effects on any of the dependent variables, while BMI yielded a significant effect on ACTH and cortisol(Table 4). Figures  1,2and 3(line graphs) shows the baseline and post-treatment values of ACTH, cortisol and *FKBP51* after adjustment for age, sex, BMI, smoking, and drinking, respectively. There were no significant changes in the expression of *NR3C1*, *HSP90*, and *POMC* from baseline to 12 weeks later (Table 4).

### Association between Δ changes in PDSS and the Δ changes in biomarkers

Table 5 shows the associations between Δchanges in the PDSS score and the Δchanges in biomarkers both before and after treatment with escitalopram. These data were analysed using multiple regression analyses with the ΔPDSS score as dependent variable and the biomarkers as explanatory variables. These analyses were adjusted for possible effects of age, sex, BMI, drinking and smoking behavior (forced entry of these possible confounders in the regression analysis). After FDR p correction, we found a significant effect of Δcortisol on ΔPDSS. No significant associations were found between the change in PDSS and the changes in the expression of the *FKBP51*, *HSP90*, *NR3C1*, and *POMC* genes, and ACTH levels (Table 5). There were no significant effects of any of those possible confounding variables, not even without FDR p correction.

## Discussion

Previous studies have shown that PD was related to abnormalities in the HPA axis, such as DNA methylation of the promoter region and polymorphisms in genes involved in the activity of HPA axis. Our previous study found that patients with PD had significantly lower levels of *FKBP5* methylation [20]. Generally, DNA hypermethylation in the promoter region suppresses gene expression, while its demethylation induces gene activation and expression [6]. Interestingly, the present study showed that *FKBP51* expression levels were significantly higher in patients with PD compared to controls, and *FKBP51* expression levels were significantly positively related to the level of cortisol and the severity of PD. Similarly, previous studies have shown that *FKBP5* expression levels are positively correlated with psychiatric disorders [25], and a higher expression of *FKBP5* was correlated with behavioral and neuroendocrine parameters [26]. The *FKBP5* gene codifies a cochaperone protein associated with modulation of the glucocorticoid receptor interaction involved in the adaptive stress response [27]. *FKBP5* could be an interesting therapeutic target for the prevention and treatment of stress-related psychiatric disorders [28].

The present study showed that the expression of the *FKBP51* decreased after 12 weeks in PD patients who have received escitalopram treatment. To our knowledge, this study is the first to investigate the effects of antidepressants on the peripheral expression of genes related to the HPA axis in patients with PD. Animal studies show that increased expression of *FKBP5* can normalize with chronic treatment with the antidepressant duloxetine [29]. Fluoxetine also significantly downregulated the expression of *FKBP5* mRNA in the prefrontal cortex [30]. And the animal study indicated that the possible mechanisms were modulation of *FKBP5* expression, GR activation, corresponding inhibition of HPA axis hyperactivity, and increase in BDNF expression [31]. Furthermore, this study revealed that *FKBP51* expression levels were significantly positively related to cortisol level. FKBP5 plays a critical role in reactivity to stress in regulation of the HPA axis function by an ultrashort negative feedback loop. FKBP5, by changing the conformation of the receptor complex, can reduce the sensitivity of NR3C1 to cortisol [32]. When cortisol binds to GR, GR can bind directly to DNA via glucocorticoid response elements and induce the expression of *FKBP5* mRNA expression [33]. However, no significant changes were found in GR, HSP90, and FKBP5 expression before and after 8 weeks of antidepressant therapy in patients with major depressive disorder [34]. Furthermore, no correlation was found between the change in PDSS and the expression of the *FKBP51* in this study. These inconsistent results may be attributed to differences in samples, follow-up time, or different antidepressants. In addition, the effect of antidepressants is related to other expression of mRNA in the treatment of PD. For example, antidepressants altered GABAA receptor subunit mRNA levels [35], and some biochemical and immune-related indicators in patients with PD [36–38]. Furthermore, a study found that miR-451a, miR-144-5p, miR-25-3p, and miR-660-5p were significantly up-regulated, miR-1, and miR-148-5p significantly down-regulated after sertraline treatment [39]. Epigenetic modifications, such as DNA methylation, post-translational histone modification, and non-coding RNA, are able to influence the expression of genes, and then play an anti-anxiety and depression effect. For example, a previous study suggests that the antidepressant mechanism of venlafaxine is partly involved in blocking effects on histone deacetylated modification and then increasing the expression of tyrosine hydroxylase and tryptophan hydroxylase [40]. However, this result was found from peripheral blood samples, and whether they reflect changes in the brain is still needed for further study. Also, previous evidence found potential regulation of *FKBP5* expression via mineralocorticoid receptor(MR) signaling, whilst *FKBP5* plays a critical role in fine-tuning the MR/GR balance [41]. Hence, future research should examine the expression of MR and aldosterone concentrations in PD and their response to escitalopram.

Also, the present study showed that the level of ACTH and cortisol decreased after 12-week in PD patients who have received escitalopram treatment, and the change in PDSS was significantly positively related to the change in cortisol levels. Similarly, patients with GAD treated with escitalopram had a significantly greater reduction in both peak and total cortisol [19]. Furthermore, some of the antidepressant and anxiolytic effects of the drug, such as Erucamide, can alleviate depression and anxiety-like behaviors in mice, and serum levels of ACTH and cortisol in mice were significantly decreased [42]. On the one hand, the HPA axis may be activated in patients with acute panic attack [43, 44], resulting in increased plasma levels of glucocorticoids. As panic attacks were controlled with medication, the activity of HPA axis decreased. On the other hand, antidepressant and anxiolytic effects of drugs suppress the HPA axis and exert antidepressant and anxiolytic-like effects [45, 46], which are associated with increased cAMP signaling and hippocampal dendritic complexity [46]. Accumulating evidence of the complex relationship between cortisol and 5-HT function, more consistent is the finding of reduced HPA function and enhanced 5-HT function on neuroendocrine challenge tests [47]. Not only that, excess cortisol can contribute to the development of anxiety by damaging hippocampal neurons [48]. An imaging study revealed that PD patients had a significantly smaller volume of the right cornu ammonis 2/3 hippocampal subfield compared to controls [49], and antidepressant treatment can counteract the reduction in hippocampal volume [50]. These results may contribute to our understanding of the effects of escitalopram on neuroendocrine hormones in PD patients. However, the influence of antidepressants on the HPA axis also depends on the duration of taking antidepressants, the type of antidepressants and the sex. For example, acute citalopram activated the HPA axis at the hypothalamic level and long-term citalopram treatment desensitized the HPA axis at the pituitary level [51]. Short-term administration of mirtazapine has immediate but only transient suppressive effects on the HPA system predominantly in women [52]. In a word, dynamic follow-up tests of HPA axis function will be necessary in the future; especially important is a longitudinal control group should be included.

The results of our study should be considered with the following limitations. First, the study did not employ a randomized controlled trial with placebo control. Second, since this was a small sample investigating the effects of escitalopram on the peripheral expression of genes related to the HPA axis and neuroendocrine hormones in patients with PD, it would be valuable to replicate our findings in a larger cohort. Third, further studies are required to investigate the DNA methylation effects of escitalopram in HPA axis-related genes for a more comprehensive analysis.

## Conclusions

The results suggest that PD may be associated with elevated levels of ACTH and cortisol, and *FKBP51* expression, and that all three biomarkers are substantially decreased in patients who have received escitalopram treatment.

**Table 1:** Demographic and clinical characteristics between PD patients and controls

Variable	PD (*n* = 77)	Controls(*n* = 82)	t/χ 2-value	p-value
**Sex, n (%)**				
Male	30(39.0)	29(35.4)	0.220	0.639
Female	47(61.0)	53(64.6)		
**Age** (years)	35.08 ± 7.76	36.04 ± 6.48	0.847	0.398
**Resident location, n (%)**				
Rural	34(44.1)	30(36.6)	0.946	0.331
Urban	43(55.8)	52(63.4)		
**Smoking history**				
Smoker, n (%)	26(33.8)	33(40.2)	0.714	0.398
Never-smoker, n (%)	51(66.2)	49(59.8)		
Smoking index	156.00 ± 88.16	175.06 ± 89.19	0.819	0.416
**Drinking (%)**				
Drinker	28(36.3)	34(41.5)	0.434	0.510
Non-drinker	49(63.6)	48(58.5)		
BMI (kg/m ^2^ )	22.67 ± 1.82	23.10 ± 3.22	1.016	0.311
Baseline PDSS score	15.61 ± 4.05			

Smoking index = daily tobacco intake×duration of smoking; BMI: Body Mass Index; PDSS: Panic Disorder Severity Scale

**Table 2:** Baseline levels of ACTH, cortisol and HPA axis related-genes expression between PD patients and controls

Variable	PD (*n* = 77)	Controls(*n* = 82)	t/Z-value	p-value
ACTH(pg/mL)	35.99 ± 13.93	20.79 ± 7.88	-8.399	0.000
Cortisol(ug/dL)	12.10 ± 4.33	8.63 ± 3.15	-5.746	0.000
NR3C1	0.025 (0.008–0.038)	0.028 (0.020–0.040)	-1.020	0.308
FKBP51	0.155 (0.086–0.237)	0.073 (0.041–0.137)	-4.701	0.000
HSP90	0.013 (0.007–0.023)	0.014 (0.008–0.016)	-0.458	0.647
POMC	0.004 (0.002–0.008)	0.003 (0.002–0.006)	-1.627	0.104

ACTH: Adreno-cortico-tropic-hormone; FKBP51: FKBP Prolyl Isomerase 51; HSP90: Heat Shock Protein 90; NR3C1: Nuclear Receptor Subfamily 3 Group C Member 1; POMC: Proopiomelanocortin

**Table 3:** Correlation among ACTH, Cortisol, severity of disease and expression levels of HPA axis-related genes in patients with PD pre-treatment

Variables	ACTH	Cortisol	PDSS	FKBP51	HSP90	NR3C1	POMC
ACTH	-	0.322^**^	0.017	0.106	-0.149	-0.067	-0.184
Cortisol		-	0.001	0.380^**^	0.053	0.142	0.114
PDSS			-	0.338^**^	0.043	0.355^**^	0.110
FKBP51				-	0.081	0.565^**^	-0.065
HSP90					-	0.053	0.049
NR3C1						-	-0.007
POMC							-

ACTH: Adreno-cortico-tropic-hormone; PDSS: Panic Disorder Severity Scale; FKBP51: FKBP Prolyl Isomerase 51; HSP90: Heat Shock Protein 90; NR3C1: Nuclear Receptor Subfamily 3 Group C Member 1; POMC: Proopiomelanocortin. ^*^p < 0.05, ^**^p < 0.01

**Table 4:** Changes in the PDSS score, peripheral HPA axis-related gene expression, and ACTH and cortisol levels in patients with PD both before and after treatment with escitalopram

	Mean (SE)		Tests of the model parameters effects			
Dependent variables	Pre-treatment	Post-treatment	Wald	df	*p*-value	FDR p values
PDSS	15.72(0.481)	9.28(0.356)	627.07	1	< 0.001	0.0018
FKBP51	0.3123(0.0596)	0.1026(0.0177)	10.19	1	0.001	0.0018
NR3C1	0.0547(0.0114)	0.0564(0.0163)	0.00	1	0.934	0.934
HSP90	0.0266(0.0041)	0.0235(0.0084)	0.10	1	0.751	0.876
POMC	0.0080(0.0012)	0.0064(0.0014)	0.66	1	0.918	0.585
ACTH pg/mL) BMI	37.96(1.55) -	21.39(0.91)	92.75 5.09	1 1	< 0.001 0.029	0.0018
Cortisol (pg/mL) BMI	12.51(0.47) -	8.77(0.38)	38.37 12.75	1 1	< 0.001 < 0.001	0.0018

ACTH: Adreno-cortico-tropic-hormone; FKBP51: FKBP Prolyl Isomerase 51; HSP90: Heat Shock Protein 90; NR3C1: Nuclear Receptor Subfamily 3 Group C Member 1; POMC: Proopiomelanocortin. FDR: False discovery rate

All results of GEE analyses, repeated measures design (with time as fixed factor), that were adjusted for age, sex, body mass index (BMI), smoking and drinking behavior. The significant confounders are shown (only BMI was significant for ACTH and cortisol)

**Table 5:** Associations between changes in severity of panic and changes in the expression of HPA axis related-genes, and ACTH and cortisol levels in panic disorder patients treated with escitalopram during 12 weeks

	ΔPDSS			
	Β	t	p-value	FDR p value
ΔACTH	0.178	1.472	0.146	0.272
ΔCortisol	0.431	3.879	< 0.001	0.006
ΔFKBP51	0.156	1.216	0.229	0.275
ΔHSP90	-0.017	-0.140	0.889	0.889
ΔNR3C1	0.167	1.351	0.181	0.272
ΔPOMC	0.223	1.927	0.058	0.174

Δ: changes in PDSS, ACTH and cortisol levels, and the expression of HPA axis related-genes from baseline to 12 weeks later. All results of multivariate regression analysis with the Δ changes in the PDSS as dependent variable and the biomarkers as explanatory variable. All results are adjusted for possible effects of age, sex, body mass index, smoking and drinking behavior by forced entry of those variables in the regression. Not one of these variables had a significant effect

ACTH: Adreno-cortico-tropic-hormone; PDSS: Panic Disorder Severity Scale; FKBP51: FKBP Prolyl Isomerase 51; HSP90: Heat Shock Protein 90; NR3C1: Nuclear Receptor Subfamily 3 Group C Member 1; POMC: Proopiomelanocortin

### Electronic supplementary material

Below is the link to the electronic supplementary material.

Supplementary Material 1

Supplementary Material 2

## Data Availability

The data used and analyzed during the current study are available from the corresponding author on reasonable request.
