# Supplementary material for: Effects of antidepressant on FKBP51 mRNA expression and neuroendocrine hormones in patients with panic disorder
Source: BMC Psychiatry. 2024 Apr 10;24:269. doi: 10.1186/s12888-024-05704-4 (PMC11005249; doi:10.1186/s12888-024-05704-4)
Supplement: Supplementary file 1 — Supplementary Material 1 [file 12888_2024_5704_MOESM1_ESM.docx]

Table S1. Primer design for validating the expression level of HPA aixs-related genes

| Gene | Primer | Primer sequence(5'—3') |
| --- | --- | --- |
| Reference gene  (GAPDH) | Forward | TGACTTCAACAGCGACACCCA |
|  | Reverse | CACCCTGTTGCTGTAGCCAAA |
| Nr3c1 | Forward | GCAGAACTGAGGCACTTAGGA |
|  | Reverse | GCCAAGGTTTCCTCCCATAG |
| Fkbp51 | Forward | TCTGGCGTGAGTTGTGAAAG |
|  | Reverse | GCCCTAGTTCAATGGTCAGC |
| Hsp90 | Forward | CACAGAAAACAGGAATGCAGACA |
|  | Reverse | ACAACGTGGACACTAAGAGAACACA |
| POMC | Forward | CCCCTGGTGACGCTGTTC |
|  | Reverse | CCCGCTGTGCCCTCACT |
