# Supplementary material for: Effects of antidepressant on FKBP51 mRNA expression and neuroendocrine hormones in patients with panic disorder
Source: BMC Psychiatry. 2024 Apr 10;24:269. doi: 10.1186/s12888-024-05704-4 (PMC11005249; doi:10.1186/s12888-024-05704-4)
Supplement: Supplementary file 2 — Supplementary Material 2 [file 12888_2024_5704_MOESM2_ESM.docx]

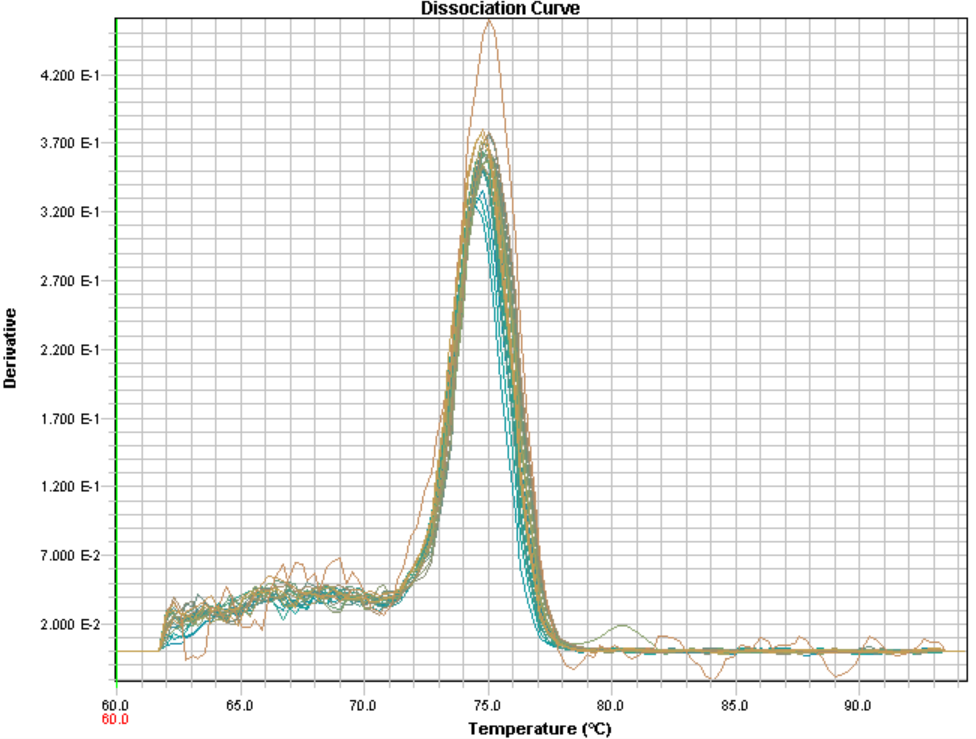


Figure S1. the melting curve of *NR3C1* gene

1
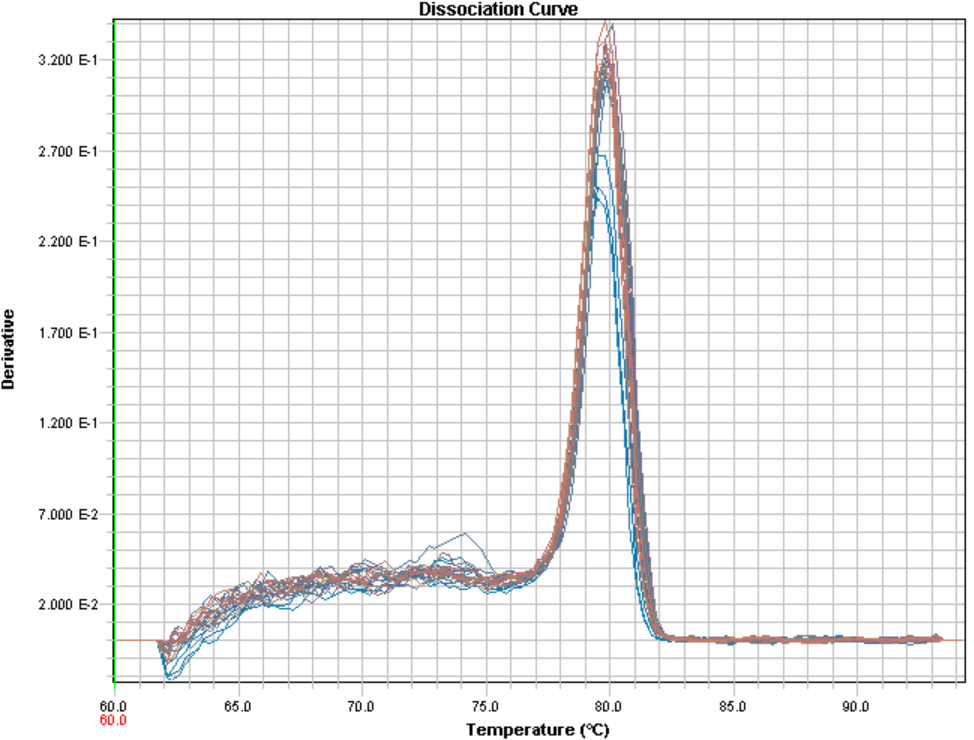
`

Figure S2. the melting curve of *FKBP51* gene


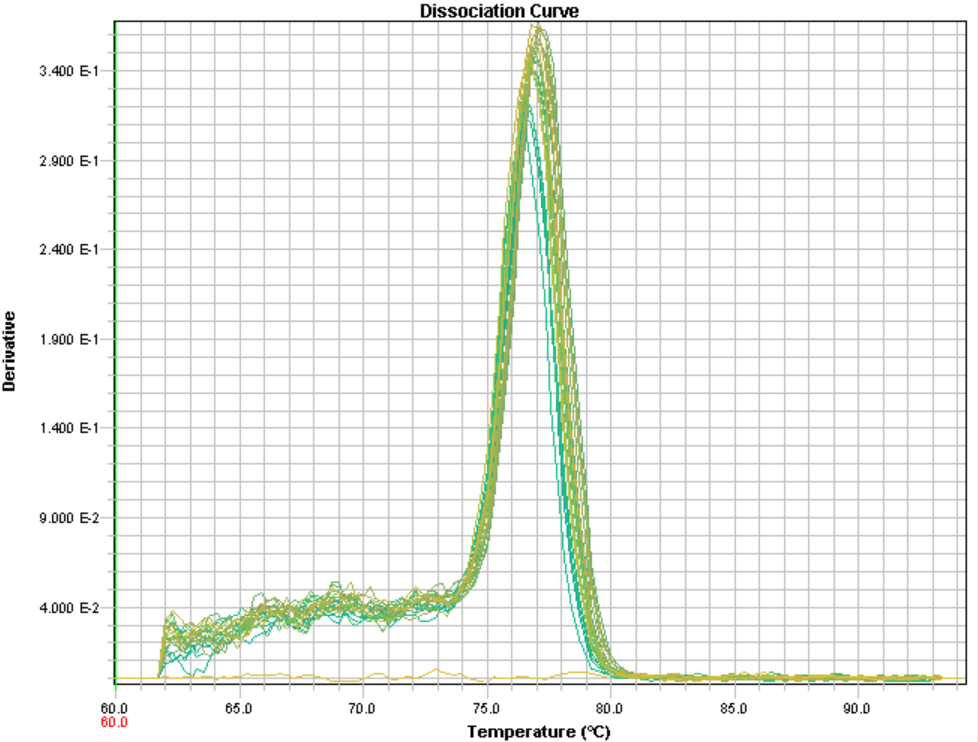


Figure S3. the melting curve of *HSP90* gene


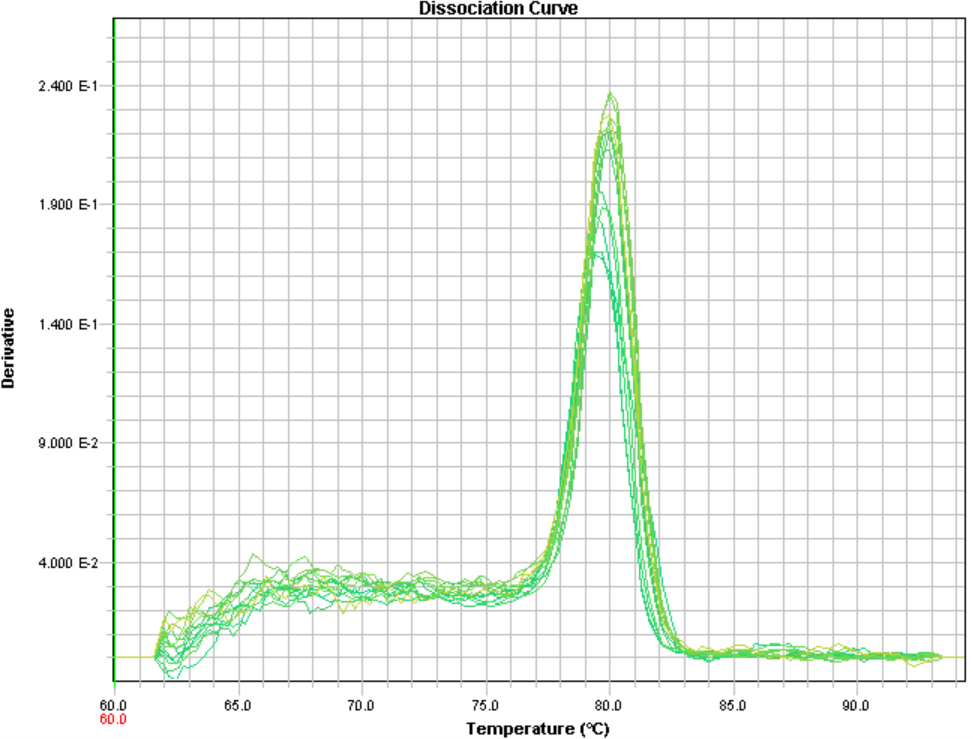


Figure S4. the melting curve of *POMC* gene
